# Genetic analysis of a pedigree with *MECP2* duplication syndrome in China

**DOI:** 10.1186/s12920-024-01831-9

**Published:** 2024-02-19

**Authors:** Lan Zeng, Hui Zhu, Jin Wang, Qiyan Wang, Ying Pang, Zemin Luo, Ai Chen, Shengfang Qin, Shuyao Zhu

**Affiliations:** 1 Department of Medical Genetics and Prenatal Diagnosis, Sichuan Provincial Maternity and Child Health Care Hospital, Chengdu, Sichuan China; 2 Department of Pediatrics, Sichuan Provincial Maternity and Child Health Care Hospital, No. 290, Sha Yan West 2Nd Road, Chengdu, 610031 Sichuan China; 3 Department of Radiology, Sichuan Provincial Maternity and Child Health Care Hospital, Chengdu, Sichuan China

**Keywords:** *MECP2*, Mental retardation, Hypoevolutism, *MECP2* duplication syndrome, Copy number variation sequencing

## Abstract

**Background:**

*MECP2* duplication syndrome (MDS) is a rare X-linked genomic disorder that primarily affects males. It is characterized by delayed or absent speech development, severe motor and cognitive impairment, and recurrent respiratory infections. MDS is caused by the duplication of a chromosomal region located on chromosome Xq28, which contains the methyl CpG binding protein-2 (*MECP2*) gene. *MECP2* functions as a transcriptional repressor or activator, regulating genes associated with nervous system development. The objective of this study is to provide a clinical description of MDS, including imaging changes observed from the fetal period to the neonatal period.

**Methods:**

Conventional G-banding was employed to analyze the chromosome karyotypes of all pedigrees under investigation. Subsequently, whole exome sequencing (WES), advanced biological information analysis, and pedigree validation were conducted, which were further confirmed by copy number variation sequencing (CNV-seq).

**Results:**

Chromosome karyotype analysis revealed that a male patient had a chromosome karyotype of 46,Y,dup(X)(q27.2q28). Whole-exon duplication in the *MECP2* gene was revealed through WES results. CNV-seq validation confirmed the presence of Xq27.1q28 duplicates spanning 14.45 Mb, which was inherited from a mild phenotype mother. Neither the father nor the mother's younger brother carried this duplication.

**Conclusion:**

In this study, we examined a male child in a family who exhibited developmental delay and recurrent respiratory tract infections as the main symptoms. We conducted thorough family investigations and genetic testing to determine the underlying causes of the disease. Our findings will aid in early diagnosis, genetic counseling for male patients in this family, as well as providing prenatal diagnosis and reproductive guidance for female carriers.

## Introduction

*MECP2* duplication syndrome (MDS) is a rare X-linked genomic disorder that was reported almost simultaneously by Meins et al. [1] and Van Esch et al. [2] in 2005. The classical phenotype primarily affects males, with 100% of male patients displaying explicit symptoms. The core phenotype of MDS patients includes developmental delay (DD), moderate to severe intellectual disability, early-onset muscle tone hypotonia, autistic features, epilepsy, delayed speech and language development, recurrent respiratory tract infections, gastrointestinal problems, and a shortened lifespan, with death often occurring before the age of 25 years [3, 4]. Although there is phenotypic variability between families, it is generally observed that the severity of phenotypes remains consistent within the same family. MDS, a condition associated with cognitive impairment and severe progressive neurological symptoms, is a significant contributor to X-linked intellectual disability in approximately 2% of males. It is worth noting that female carriers exhibit fewer clinical phenotypes, which can be attributed to the protective mechanisms of non-random inactivation of the X chromosome [3, 5–7]. In this study, we investigated a male child with developmental delay and recurrent respiratory tract infections as the main symptoms. The child was suspected to have *MECP2* duplication syndrome, and genetic analysis was conducted to identify the underlying causes. To more accurately describe the complete clinical spectrum of MDS, we have included fetal brain MRI images of patients with MDS, as well as the rare stenosis bronchus phenotype.

## Materials and methods

### Ethical approval

The study was approved by the Institutional Ethics Committee of Sichuan Provincial Maternity and Child Health Care Hospital (protocol #20,210,331–028).

### Sample collection

Clinical data were collected from a family of patients with recurrent variants of the *MECP2* gene. Blood samples were obtained from the proband, their parents, their uncle, and their maternal grandmother and stored at –20 °C.

### Exome sequencing

Genomic DNA was extracted from peripheral blood leukocytes using the QIAamp DNA Blood Mini Kit (Dusseldorf, Germany) following the manufacturer's instructions. The purity and quality of the DNA were assessed using NanoDrop and agarose gelelectrophoresis, respectively. The genomic DNA was then sheared into random fragments and purified. For sequencing the exomes of the patient and her parents, trio-WES technology (Mygenostics, Beijing) was employed. The xGen Exome Research Panel v4.0 whole-exon capture chip from IDT (Intelligent Data Termina) was used for genome-wide exon capture. After passing the quality control, the captured fragments were used for genomic library preparation and subsequently sequenced on the Applied Biosystems 3730xl DNA Analyzer. The average sequencing depth was 100 × , and more than 98% of the area achieved a coverage of more than 20 × .

### Chromosome karyotype analysis and copy number variation sequencing

Karyotyping was performed following the International Nomenclature System of Human Cytogenetics (ISCN2020) standard, using a fully automated scanning system (Zeiss, Germany). The 20 split phases were counted, and 5 karyotypes were analyzed. Chromosome karyotype chimerism increased the count to 50 split phases. Genomic DNA (10 ng) was fragmented, the DNA library was constructed, and low-depth high-throughput sequencing (Chigene Medical Laboratory, Beijing, China) was performed. The sequencing data were compared to the hg19 reference genome, and chromosomal aneuploidy variation and copy number variations (CNVs) above 100 kb were recorded and analyzed. The analysis of CNVs primarily involves the use of databases such as the Database of Genomic Variants (DGV, http://dgv.tcag.ca/dgv/app/home), the Database of Genomic Variation and Phenotype in Humans (DECIPHER, https://decipher.sanger.ac.uk), and the Online Mendelian Inheritance in Man (OMIM, https://omim.org). Additionally, the gene dosage effect database ClinGen (https://www.ncbi.nlm.nih.gov/projects/dbvar/clingen) was consulted to analyze the significance of chromosomal deletions or duplications.

### Mutation validation and cosegregation analysis

To identify potential causes, we utilized whole-exome sequencing and conducted in-depth mutation analysis. Clinical symptoms were compared using various databases and literature sources, including OMIM, HGMD(https://www.hgmd.cf.ac.uk/), ClinVar (nih.gov), MITOMAP (http://www.MITOMAP.org), and PubMed (nih.gov). Candidate gene variants were verified in families. The identified sequence variant was further interpreted and classified according to the guidelines provided by the American College of Medical Genetics and Genomics (ACMG) [8].

## Results

### Case presentation

**Fig. 1:**
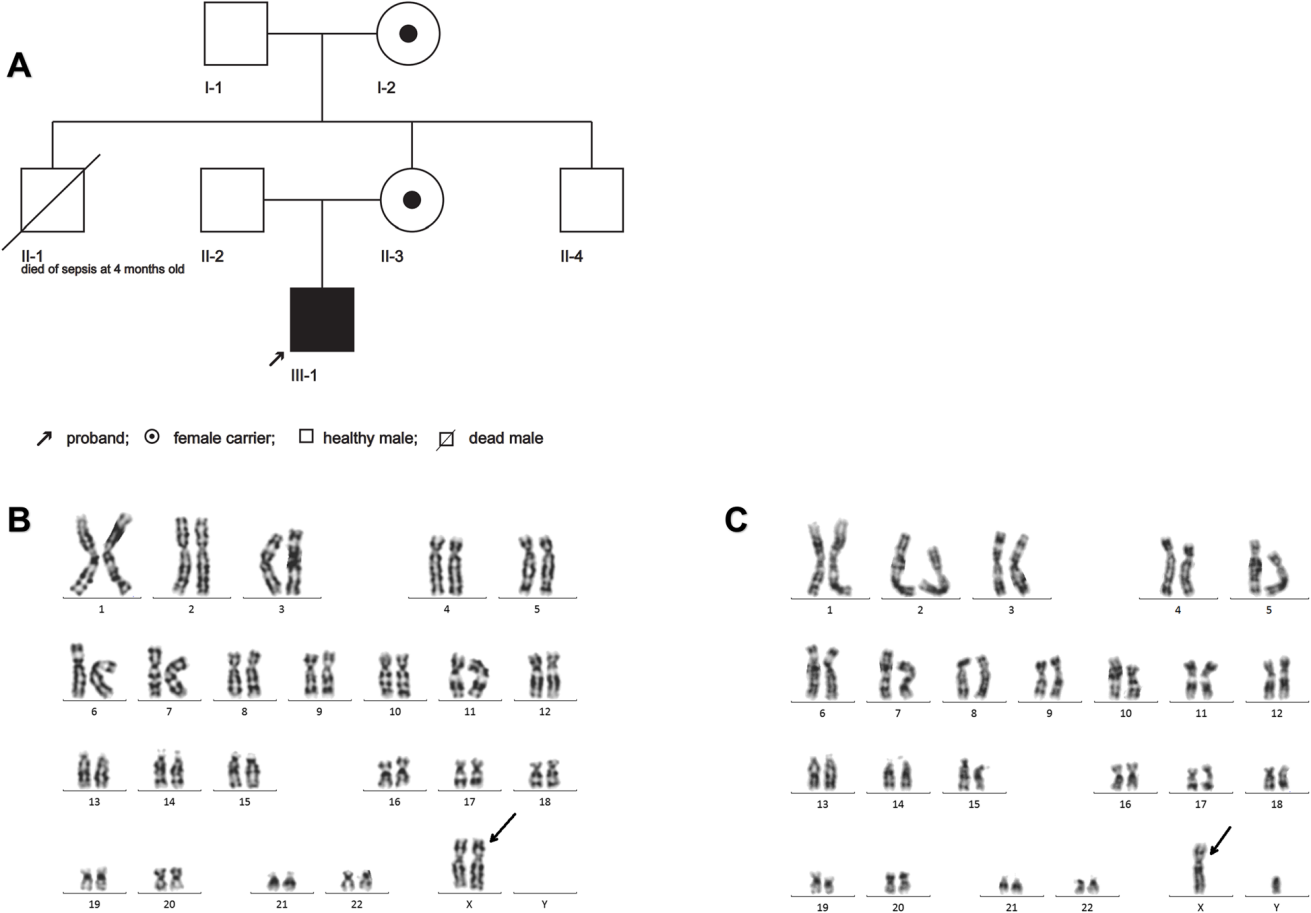
**A** Pedigrees of MDS; **B** Karyotype analysis of the proband's mother 46,X,dup(X)( q27.2q28), black arrow indicating the site of duplication; **C** Karyotype analysis of the proband 46,Y,dup(X)(q27.2q28),  black arrow indicating the site of duplication

This study recruited a total of five family members from the same Chinese family (Fig. 1A).

**Fig. 2:**
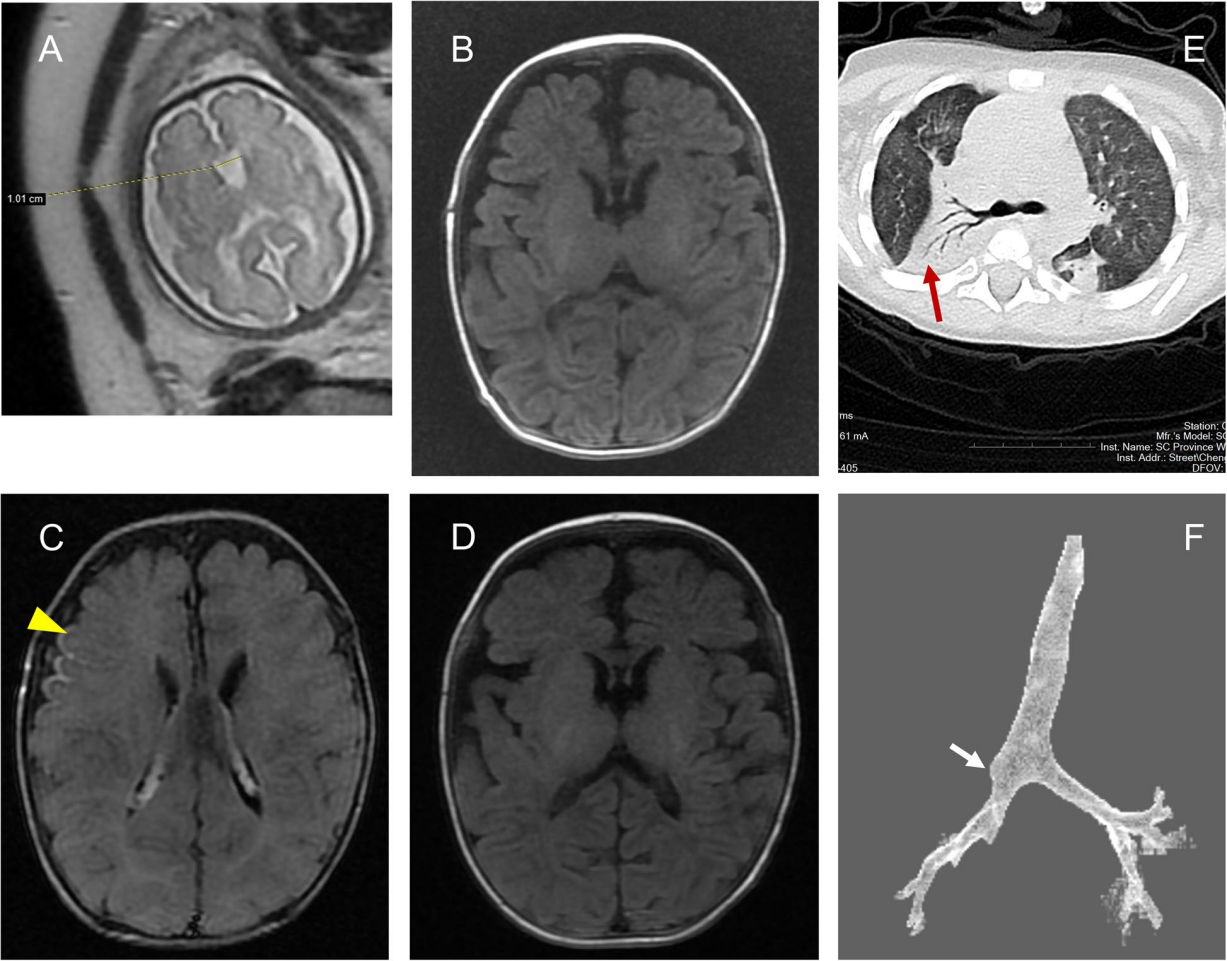
Brain MRI and chest CT images of the proband; **A** Fetal brain MRI at 30 + 6 weeks suggesting a slight widening of the Cavum septum pellucidum; **B** At 1 month and 30 days of birth, T1WI showed that high signals were faintly visible in the posterior parts of the hindlimbs of bilateral internal capsules; **C** At 1 month and 30 days old, T2-FLAIR delayed enhancement suggested right frontal pia enhancement, as indicated by the yellow arrow; **D** At the age of 3 months, T1WI showed high signals in the posterior limbs of bilateral internal capsules, but not in the forelimbs of internal capsules; **E** Chest CT indicates inflammation. Red arrows indicate lung consolidation; **F** 3D reconstruction of the airway showing a suspected stenosis of the bronchus of the right superior lobe indicated by the white arrow

III-1 was a male newborn, aged one month and 25 days, non-consanguineous parents. Initially, he was admitted to our pediatric neurology department due to recurrent fever and poor response, with suspicion of meningitis. Prenatal ultrasound and MRI were conducted at 30 + 6 weeks of fetal cavity septum pellucidum width anomaly (Fig. 2A). Fetal echocardiography revealed the presence of a ventricular septal defect. The pregnant woman underwent prenatal diagnostic consultation but declined amniotic fluid testing to further confirm the diagnosis. The baby was delivered by cesarean section at 39 weeks and 2 days of gestation due to fetal distress. Electronic fetal monitoring showed variable deceleration. The amniotic fluid exhibited grade III fecal contamination at birth. No abnormalities were observed in the umbilical cord or placenta. The Apgar scores were 10, 10, and 10. The child had a birth weight of 2625 g (< 3th percentile), a birth length of 49 cm (10th to 25th percentile), and a birth head circumference of 33 cm (25th percentile). The child was diagnosed with neonatal pneumonia due to breathing difficulties 20 h after birth. Additionally, the child was diagnosed with neonatal hyperbilirubinemia 11 days after birth, and with neonatal sepsis due to fever and lethargy within 25 days after birth, resulting in three hospitalizations. During hospitalization, a physical examination revealed the following: temperature 38.2 °C, heart rate 148 beats/min, respiratory rate 55 times/min, weight 2.8 kg (< 3th percentile), length 50 cm (< 3th percentile), head circumference 34 cm (< 3th percentile), anterior fontanelle is tense and swollen, depressed nasal bridge, high palate, macrotia, low-set ears, infantile muscular hypotonia, and cryptorchidism. Blood routine analysis showed white blood cell 17.8 × 10^9^ (reference range 5.6–15 × 10^9^), neutrophil 15.8 × 10^9^ (reference range 3.2–10.7 × 10^9^), and C-reactive protein 64 mg/L (reference range 0–10 mg/L). Cerebrospinal fluid (CSF) white blood cell counts 164 × 10^6^/L (reference range 0- 5 × 10^6^). CSF glucose levels 2.1 mmol/L (reference range 2.5–4.5 mmol/L), CSF-to-blood glucose ratios 0.36 (reference range 0.44–0.90). The CSF culture was negative and no pathogenic microorganisms were found by CSF next generation sequencing. His brain MRI revealed delayed myelination (Fig. 2B and D) and leptomeningeal enhancement (Fig. 2C). No abnormalities were observed in cellular immune markers or humoral immunity. The patient experienced recurrent seizures during the course of the disease, specifically generalized tonic–clonic seizures (GTCS). The video electroencephalogram did not reveal any abnormalities. Additionally, there was consolidation with atelectasis in the right upper lobe (Fig. 2E). No abnormalities were found in the blood ammonia, blood gas analysis, dried blood spots of tandem mass spectrometry, or urine organic acid analysis. Following a 14-day course of anti-infective treatment with ceftriaxone and vancomycin, the cerebrospinal fluid cytological examination returned to normal, and the lung inflammation improved, leading to the patient's discharge.

Regular follow-up in the Department of Child Health Care revealed the following milestones: the patient was able to achieve head control at 6 months, sit steadily without support at 12 months of age, and had delayed speech and language development. A child healthcare physician utilizes a modified version of the Gesell Development Schedules, which have been standardized in China, to evaluate the child. The patient's developmental quotient was found to be 43, indicating a moderate developmental deficiency [9]. Over a period of 2 years and 7 months, the patient experienced pneumonia 7 times, with 2 of those instances believed to be related to gastroesophageal reflux. After undergoing cryptorchidism surgery, the patient developed a severe hospital-acquired infection by fever and shortness of breath, ultimately leading to a diagnosis of severe pneumonia. Chest CT and flexible bronchoscopy revealed stenosis of the bronchus of the right superior lobe (Fig. 2F).

As the patient grew older, the gastroesophageal reflux improved, but he developed chronic constipation and sleep abnormalities.

II-3, a 23-year-old woman and mother of proband III-1, is the second child in a healthy unrelated family. She does not have any mental or developmental retardation. However, she does have midface retrusion.

Additionally, she is experiencing difficulties in conceiving. II-1, the older brother of II-3, was delivered vaginally and had no abnormalities at birth. He developed a recurring fever shortly after birth and passed away due to septicemia at approximately 4 months old. Unfortunately, we were unable to obtain a biological sample from him for testing.

I-2, the maternal grandmother of III-1, possesses the same duplicate variant of *MECP2* as II-3. However, she does not exhibit any clinical symptoms.

### Genetic and bioinformatics analysis

**Fig. 3:**
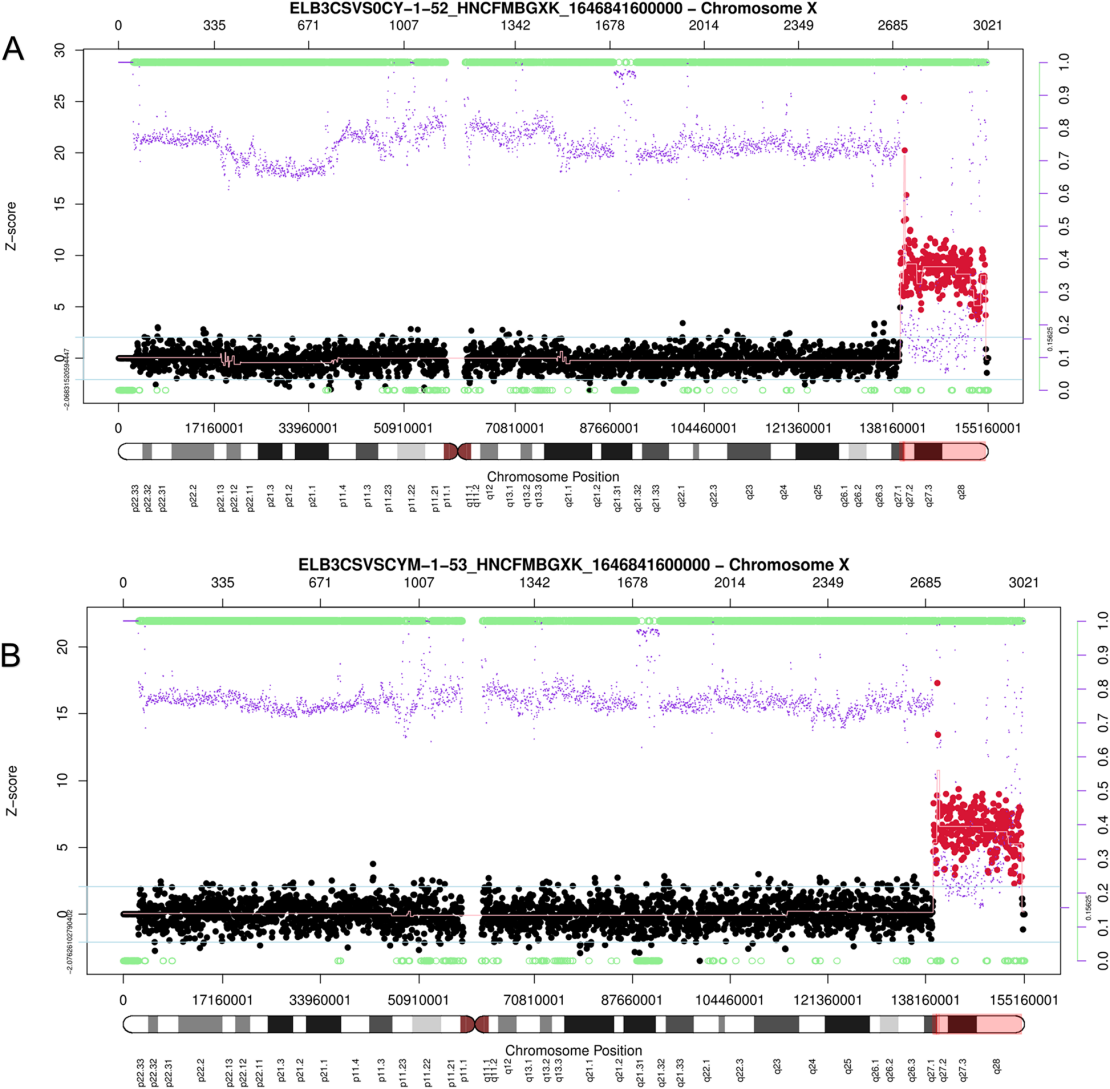
CNV-seq results of the proband and the proband's mother, with the red area indicating duplication regions. **A** The proband; **B** The proband's mothe

By chromosomal karyotype analysis, there was no abnormal karyotype of I-1, II-2 or II-4. The abnormal karyotype of I2 indicates Xq27.2-q28 duplication, and the karyotype of II-3 suggested that she inherited her mother's Xq27.2-q28 duplication (Fig. 1C). The chromosome karyotype of the patient (III-1) suggested a male and inherited a maternal Xq27.2-q28 duplication (Fig. 1D). The CNV-seq results showed that the breakpoint of the duplication position was located between 140,360,001 and 154,810,000, spanning 14.45 Mb (Fig. 3A). The results of I-2 and II-3 showed that the breakpoint of the duplication position was located between 139,460,001 and 154,810,000, spanning 15.35 Mb, as female carriers of the *MECP2* duplication (Fig. 3B).

The repetition region contained 126 protein-coding genes, including *MECP2*, GDP dissociation inhibitor 1 gene (*GDI1*, OMIM # 300,104), and member RAS oncogene family (*RAB39B*, OMIM # 300,774). However, no similar reports were found in the DGV database. Several pathogenic cases have been reported in the DECIPHER and ClinVar databases. Regional analysis of OMIM disease-causing genes with duplications range comprising at least 38 genes: *ABCD1*, *AFF2*, *ATP2B3*, *BCAP31*, *CCNQ*, *EMD*, *FLNA*, *HCFC1*, *IDS*, *L1CAM*, *MECP2*, *OPN1LW*, *RAB39B*, *RPL10*, *SOX3*, *TMLHE*, *IRAKl*, etc. According to the clinical manifestations of the child, combined with the results of the CNV-seq analysis, MDS was confirmed.

## Discussion

*MECP2* duplication syndrome (MDS, OMIM #300,260) is a rare neurological disorder that occurs in 1 in 150,000 male live births [10]. In the past 20 years, there have been nearly 200 cases of MDS worldwide. However, the understanding of the disease in China is still limited, with only over 20 MDS pedigrees reported. The cause of MDS is the duplication or triplication of the chromosomal region containing the methyl CpG binding protein-2 (*MECP2*) gene (OMIM #300,005). The *MECP2* gene is located in the q28 region of the X chromosome. It is a multifunctional gene that acts as a transcriptional regulator and chromatin remodeler, playing a role in nervous system development. Sanmann JN et al. [11] discovered a region of overlap between a region of overlap spanning from 79.7 kb to 15.5 Mb. This region contains multiple genes, including *MECP2*, interleukin-1 receptor-associated kinase 1 (*IRAKl*, OMIM #300,283), filamin Aalpha (*FLNA*, OMIM #00017), and L1 cell adhesion molecule (*L1CAM*, OMIM #08840), all of which are crucial for human neural development. The smallest reported region of overlap (~ 79.7 kb) contains only the *MECP2* and *IRAKl* genes [12].

The MECP2 protein is a multifunctional protein primarily responsible for transcriptional regulation. It is involved in various processes, such as methylation, phosphorylation, ubiquitination, and acetylation, which contribute to the epigenetic inheritanceof DNA [12]. Additionally, it plays a role in regulating chromatin structure, gene expression, RNA splicing, and miRNA processing, all of which are crucial regulatory factors in the nervous system [12, 13].

The patient initially considered immunodeficiency-related diseases due to recurrent infections, but the results of cellular and humoral immunity do not support this hypothesis. During this hospitalization, the patient experienced recurrent seizures, accompanied by a decrease of more than 0.40 in CSF-to-blood glucose ratios. The child was suspected of having glucose transporter 1 deficiency syndrome (GLUT1DS1, OMIM # 606,777); however, seizures were secondary to meningitis. After retrospectively analyzing the family history of this patient, medical genetics experts suspected that the child may have an X-linked genetic disease. Following sufficient communication with the patient’s parents, simultaneous chromosome and Trio-WES were requested to avoid repeated blood sampling. We utilized the ClinGen dosage sensitivity evaluation system to assess genes located between 140,360,001 and 154,810,000 repeats. Our evaluation identified three significant regions of Triplosensitivity: The Xq28 region (X:153,273,980–153375749; including *MECP2*), the Xq28 recurrent region (X:153,624,564–153,783,898; including *GDI1*), and the Xq28 recurrent region (int22h1 / int22h2-flanked) (X:153,624,564–153,783,898; including *RAB39B*). The Triplosensitivity score for these three repeat regions is 3 points, indicating Sufficient Evidence that an increase in the copy number of these genes will result in abnormal physiological functions [14]. According to Technical standards for the interpretation and reporting of constitutional copy-number variants: a joint consensus recommendation of the American College of Medical Genetics and Genomics (ACMG) and the Clinical Genome Resource (ClinGen), the patient's copy-number variant was evaluated as “Pathogenic” [15]. Notably, *GDI1* has been associated with Intellectual developmental disorder, X-linked 41 (OMIM # 300,849), and *RAB39B* has been linked to Intellectual developmental disorder, X-linked 72 (OMIM # 300,271) and Waisman syndrome (OMIM # 311,510). The patient's repeat contains all three of these regions, which can account for all of their clinical symptoms. We also excluded three heterozygote variants *NGLY1* (NM_018297) c.1231C > T (p.R411X), *SGO1* (NM_001199252) c.1472 + 1G > C (splicing), and *TMEM237* (NM_001044385) c.554-3_554-2 insT (splicing) that were inherited from healthy fathers. These variants were not associated with any clinical phenotype and are considered biallelic pathogenic variants. Finally, CNV-seq validation was selected based on the results of chromosome and Trio-WES.

The results indicate that the ultimate cause of recurrent infection and developmental delay in this patient was the duplication involving *MECP2*. The diseases related to protein gain of function (GOF) caused by *MECP2* predominantly affect males, accounting for approximately 90.3% of the total cases [16]. Approximately 80% of MDS variants are inherited from asymptomatic mothers. However, it is important to note that these duplications, ranging in size, can undergo changes during inheritance [17, 18]. This is due to the Xq28 region being rich in low-copy sequences, making the genome structure in this region unstable and susceptible to rearrangement. Furthermore, the size of the rearranged fragments can vary, even within the same family, leading to inconsistency in fragment sizes. If the duplication involving *MECP2* is only found in the progeny's DNA and not in the maternal DNA, it is crucial to consider the possibility of maternal reproductive chimerism. In a very small number of patients, those with *MECP2* triplets (MTS) exhibit more severe symptoms and shorter lifespans compared to individuals with MDS [3]. The relationship between the size of duplication and the severity of clinical symptoms has been subject to speculation.

**Fig. 4:**
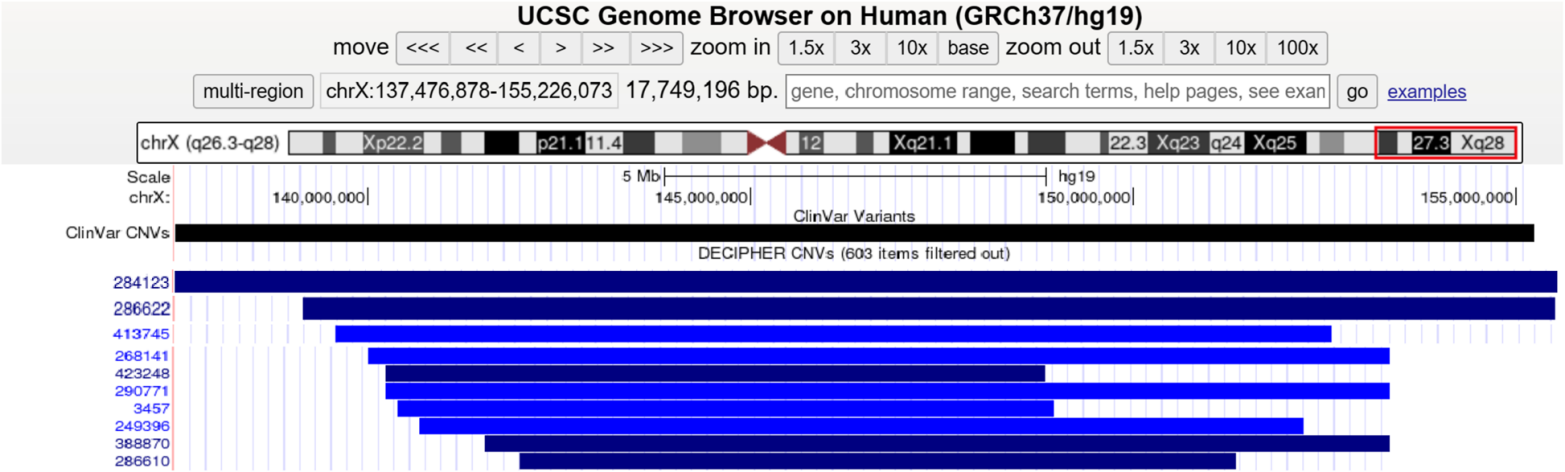
Genomic locations of 10 duplications based on the results obtained in the Table 1. The annotation is based on GRCh37/hg19

Approximately 74% of MDS patients experience respiratory infections, and many patients also have asthma and bronchomalacia [19]. In 2022, Sugitate R [20] performed laryngotracheal separation in three MDS patients with predominantly aspiration pneumonia, which effectively reduced their recurrent respiratory tract infections. Interestingly, our patient was found to have tracheal stenosis during bronchoscopy and CT, a finding that has not been reported in other cases of MDS in China. It is unclear whether Xq28 duplication correlates well with stenotic bronchus in our study, and more evidence is needed to support this hypothesis. In Table 1 (Table 1), we summarized the characteristics of cases with similar duplication regions in the DECIPHER and ClinVar databases compared to our case (Fig. 4).

**Table 1:** Comparison of genotype–phenotype of patients with Xq28 duplication syndrome

**Serial number**	**Sex**	**Location (**GRCh37)	**Pathogenicity**	**Size**	**Type**	**Phenotype(s)**
**DECIPHER Patient**						
284,123	46,XX	X:137,476,878–155,226,073	Pathogenic	17.75 Mb	Duplication	IGR, Micrognathia
286,622	46,XX	X:139,089,231–154929220	Likely pathogenic	15.84 Mb	Duplication	GDD, Microcephaly, Mild IGR, Severe short stature
290,771	46,XX	X:140,222,659–155270560	Uncertain	15.05 Mb	Duplication	NA
319,602	46,XX	X:138,802,717–153690960	pathogenic	14.89 Mb	Triplication	Premature ovarian insufficiency
268,141	46,XX	154,271,972–155,207,180	NA	935.21 Kb	Duplication	NA
473,075	46,XX	X:145,303,603–154225379	pathogenic	8.92 Mb	Duplication	NA
388,870	46,XY	X:141,522,614–154,929,412	pathogenic	13.41 Mb	Duplication	Cerebral palsy, ID, moderate, Short stature
413,745	46,XY	X:139,510,049–152101421	NA	12.59Mb	Duplication	Cryptorchidism, Delayed speech and language development, Hypospadias, ID, Moyamoya, Obesity, Secundum atrial septal defect, Short stature
249,396	46,XY	X:140,664,743–152216545	NA	11.55 Mb	Duplication	Cryptorchidism, Hypertelorism, Hypotonia, ID, Low-set ears, Micrognathia, Short stature
286,610	46,XY	X:141,977,992–151,335,231	Pathogenic	9.36 Mb	Duplication	Decreased testicular size, Hypospadias
423,248	46,XY	X:140,222,631–148838733	Pathogenic	8.62 Mb	Duplication	GDD, Recurrent infections, Strabismus
3457	46,XY	X:140,384,198–148971029	NA	8.59 Mb	Duplication	Delayed speech and language development, Feeding difficulties, ID, Medial flaring of the eyebrow, Microcephaly, Small scrotum, Strabismus
**ClinVar database**						
58,688	NA	X: 139,308,651–54917042	Pathogenic	15.5Mb	Duplication	NA
58,691	NA	X: 139,527,393–53,832,724	Pathogenic	14.2Mb	Duplication	NA
146,621	NA	X: 139,865,555–54,785,891	Pathogenic	14.8Mb	Duplication	NA
73,287	NA	X: 140,254,480–154929279	Pathogenic	14.5Mb	Triplication	NA

*IGR* intrauterine growth retardation, *GDD* global developmental delay, *ID* intellectual disability, *NA* not applicable

We found that some females carrying the duplication had intrauterine growth retardation (IGR) and were after adulthood with short stature. One female with the genotype MTS had a phenotype of premature ovarian insufficiency. In this study, the mother of the patient showed only dysplasia of the mid-face bones, and the maternal grandmother had no abnormalities. These phenotypic differences may be related to the stochastic nature of X-chromosome inactivation (XCI) [18]. Unfortunately, our laboratory was not equipped to monitor XCI at that time, but it will be one of the focuses of our subsequent extension study. More than 1,000 cases related to *MECP2* mutations have been included in HGMDpro, with approximately 23% of cases involving total gene duplication or deletion. Loss-of-function mutations in the *MECP2* gene are recognized as Rett syndrome (RTT) (OMIM #312,750) [21]. Approximately 92% of the affected individuals are women. The main clinical manifestations observed included Global developmental delay (GDD), absent speech, high-pitched cry limb apraxia, stereotypical hand wringing, microcephaly, seizures, and between 6 and 18 months of age [22]. It is important to note that due to the process of X-chromosome inactivation in females, the expression of the non-mutant or mutant *MECP2* allele can vary in each cell, resulting in some retention of full-length *MECP2*. As a result, the severity of symptoms associated with Rett syndrome can vary greatly among affected individuals. In our other research projects, we encountered a unique case of a male individual with a missense variant in *MECP2* (p. Pro431Leu) who presented with autism and GDD. Unfortunately, we were unable to obtain a sample from the mother, which hindered our ability to assess the pathogenicity of the alteration.

The disease currently lacks a specific treatment but focuses on managing infections, epilepsy, and other symptoms. Prenatal diagnosis poses challenges due to the low expression of the *MECP2* gene in early embryos, with its peak expression occurring after birth, particularly in mature neurons [23]. Given that the disease primarily affects asymptomatic carriers in females, the impact on males is significant. We emphasize the significance of prenatal testing for fetuses with structural abnormalities and reaffirm that karyotyping and CNV are still the preferred methods of prenatal diagnosis for such cases. These methods are both cost-effective and irreplaceable. While whole exome sequencing (WES) has shown remarkable advancements in certain genetic areas, it has its limitations and should only be considered as an additional test when a definitive diagnosis cannot be obtained through the aforementioned methods [24]. Therefore, clinicians should choose individualized detection methods for patients based on more clinical manifestations and multidisciplinary cooperation such as genetics and imaging to obtain better diagnostic capabilities. As a result, genetic counseling becomes crucial for families who have children with MDS.

## Conclusions

We present a neonatal case of MDS with recurrent multiple organ infections. We performed CNV-seq sequencing and discovered that the patient and some female family members had approximately 10 Mb in the Xq28 region, which encompassed the entire *MECP2* gene. Given the variable clinical phenotype of MDS and the lack of clinical manifestations in most female carriers, prenatal diagnosis poses a challenge. Establishing family follow-up centers could be an effective approach to prevent birth defects.

## Data Availability

The datasets generated and analysed during the current study are available in the ClinVar repository, SCV003853606.
